# Tobacco Smoking Using Midwakh Is an Emerging Health Problem – Evidence from a Large Cross-Sectional Survey in the United Arab Emirates

**DOI:** 10.1371/journal.pone.0039189

**Published:** 2012-06-15

**Authors:** Mohammed Al-Houqani, Raghib Ali, Cother Hajat

**Affiliations:** 1 Faculty of Medicine and Health Sciences, UAE University, Al-Ain, United Arab Emirates; 2 Cancer Epidemiology Unit, University of Oxford, Oxford, United Kingdom; 3 Public Health & Research Department, Health Authority of Abu Dhabi, Abu Dhabi, United Arab Emirates; Fundación para la Prevención y el Control de las Enfermedades Crónicas No Transmisibles en América Latina (FunPRECAL), Argentina

## Abstract

**Introduction:**

Accurate information about the prevalence and types of tobacco use is essential to deliver effective public health policy. We aimed to study the prevalence and modes of tobacco consumption in the United Arab Emirates (UAE), particularly focusing on the use of Midwakh (Arabic traditional pipe).

**Methods:**

We studied 170,430 UAE nationals aged ≥18 years (44% males and 56% females) in the Weqaya population-based screening program in Abu Dhabi residents during the period April 2008–June 2010. Self-reported smoking status, type, quantity and duration of tobacco smoked were recorded. Descriptive statistics were used to describe the study findings; prevalence rates used the screened sample as the denominator.

**Result:**

The prevalence of smoking overall was 24.3% in males and 0.8% in females and highest in males aged 20–39. Mean age (SD) of smokers was 32.8 (11.1) years, 32.7 (11.1) in males and 35.7 (12.1) in females. Cigarette smoking was the commonest form of tobacco use (77.4% of smokers), followed by Midwakh (15.0%), shisha (waterpipe) (6.8%), and cigar (0.66%). The mean durations of smoking for cigarettes, Midwakh, shisha and cigars were 11.4, 9.3, 7.6 and 11.0 years, respectively.

**Conclusions:**

Smoking is most common among younger UAE national men. The use of Midwakh and the relatively young age of onset of Midwakh smokers is of particular concern as is the possibility of the habit spreading to other countries. Comprehensive tobacco control laws targeting the young and the use of Midwakh are needed.

## Introduction

Tobacco use is the single greatest cause of preventable death in the world today, killing 6 million people every year with a projected increase to 8 million per year by 2030 [1]. It is a major risk factor for most chronic diseases including cardiovascular disease, stroke, chronic respiratory diseases and a large number of cancers [2], [3]. Manufactured cigarettes are the most common type of tobacco consumed worldwide but other modes (such as chewing tobacco in South Asia and Shisha (or waterpipe) smoking in the Middle East) are also common in particular regions [1].

Although tobacco was introduced to the Arab world more than 500 years ago, consumption has increased rapidly in the last 50 years with the convenience and affordability of manufactured cigarettes. Male smoking rates in the Arab world are amongst the highest in the world, with a prevalence of up to 77% [1]. Smoking is generally still uncommon in women in the Arab world [4]. Other forms of tobacco consumption are also common in some Arab countries, particularly the smoking of shisha (water-pipe) which has also increased significantly over the last 30 years [5].

**Figure 1:**
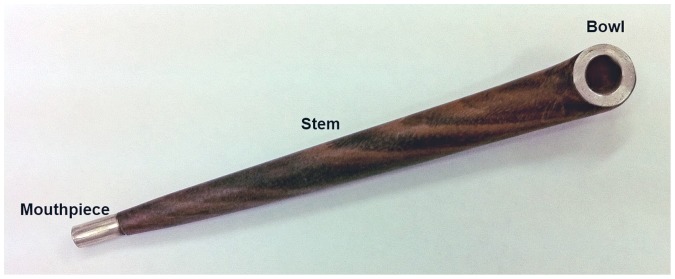
An example of the ‘Midwakh’ – a small pipe for smoking tobacco of Arabian origin.

Midwakh is a small pipe for smoking tobacco of Arabian origin (Figure 1). It was traditionally smoked by the Bedouin and sailors in the UAE. The Midwakh bowl is small and is filled with ∼0.5 grams of dry tobacco (Dokha) for each use. Typically smokers would require only 2 inhalations to fully burn the Dokha before it needs to be re-filled. There have been some reports that that the use of the Midwakh is increasing in the Gulf and particularly in the United Arab Emirates [6], [7] but to date there have been no comprehensive, population-based surveys of the use of Midwakh in any country and the WHO surveys (GSHS, GYTS and GTSS) in the region have not reported its use [8]–[10]. There is also nothing in the published literature about how Midwakh compares to cigarettes in term of its addictiveness and health effects but it is likely to be equally harmful and as with any tobacco product, it will increase the risk of many chronic diseases, which are already increasing rapidly in the UAE and the region [11].

This paper presents the first evidence of the prevalence of Midwakh smoking (as well as cigarette and shisha smoking) from a large population based cross-sectional survey in the Emirate of Abu Dhabi as part of the Weqaya screening program for cardiovascular risk factors for all adults in Abu Dhabi [11], [12].

## Methods

### Ethics Statement

This study was approved by Al-Ain medical district human research ethics committee – protocol No. 11/28. A written consent was obtained by trained nursing staff in line with the principles of the Abu Dhabi Medical Research Council which regulates human health research in Abu Dhabi and the Health Authority Abu Dhabi (HAAD) consent policy.

The Weqaya Cardiovascular Program screened UAE nationals aged ≥18 years linked to the provision of a comprehensive health insurance plan [12]. This study included UAE nationals screened during the period April 2008–June 2010. Further details of the screening program are available elsewhere [11], [12]. The screening was carried out by a network of 25dedicated primary care clinics across the emirate of Abu Dhabi. A total of 173,501 adults were screened (44% males and 56% females) comprising approximately 94% of adult Emiratis residing in Abu Dhabi [13] A comparison of the Weqaya study cohort with the Abu Dhabi population demographic reveals under-representation of only the age group 20–24 in the Weqaya cohort, this age group represents 15.6% (95% CI: 15.1–16.1%) in the Weqaya cohort and 20.9% (95% CI: 20.7–21.1%) of the population demographic.

Individuals' tobacco smoking habits were ascertained by self-report with questions being interviewer administered. Questions were asked on smoking status (current vs. nonsmokers), type (cigarette, Midwakh, cigars and shishas) quantity, and duration of tobacco smoked. Current smokers were defined as smoking at least 1 cigarette per day or 1 pipe per day or 1 cigar per week for the past 12 months [14], [15]. Current shisha smoking was defined as smoking at least one shisha per month for the past 3 months [16].

We categorized subjects by age and gender and type of tobacco smoking. The prevalence of tobacco smoking was calculated as the number of smokers divided by the number of individuals in the screening program during the study period. The prevalence of each type of tobacco was calculated by multiplying the proportions of smokers of each type of tobacco by the prevalence of smoking in the whole population. The age stratified smoking prevalence was calculated as the number of smokers within an age group, divided by the contemporary study population within that age group. The proportion and the prevalence of each type of tobacco product may include subjects who smoked more than one type of tobacco products.

All statistical analyses were conducted using STATA version 10.0 (STATACorp LP, Tx, USA).

Frequencies were compared using means and standard deviations. Crude prevalence rates used the screened sample as the denominator. Confidence intervals of 95% and p less than or equal to 0.05 were taken as indicative of statistical significance.

## Results

Of the 173,501 individuals who were screened during the study period, 170, 430 (98%) had information recorded on smoking status and of these, 18,814 (11.0%) reported to be active smokers (any type of tobacco use). Table 1 presents the number of smokers categorized by gender and age group. The prevalence of smoking was much higher in men (24.3%) than in women (0.8%). Table 1 also shows the age stratified smoking prevalence rate which was highest amongst male smokers aged 20–39 and lower in older age groups.

**Table 1:** Age and gender specific prevalence of smoking.

Variables	Study population			Men			Women		
Age group	Number	smokers	Prevalence	Number	Smokers	Prevalence	Number	Smokers	Prevalence
Total	170430	18814	11.0%	74421	18045	24.2%	95678	737	0.8%
18–20	7201	551	7.7%	3187	525	16.5%	3996	22	0.6%
20–29	68209	8439	12.4%	29858	8182	27.4%	38243	243	0.6%
30–39	45690	5783	12.7%	19648	5542	28.2%	25965	235	0.9%
40–49	22320	2315	10.4%	9524	2178	22.9%	12751	134	1.1%
50–59	14276	1048	7.3%	5970	977	16.4%	8267	67	0.8%
60–69	8363	494	5.9%	4098	464	11.3%	4237	29	0.7%
70–79	3518	151	4.3%	1737	145	8.3%	1768	6	0.3%
≥80	853	33	3.9%	399	32	8.0%	451	1	0.2%

Table 2 demonstrates the proportion of different types of tobacco smoking. Cigarette smoking was the commonest form of tobacco use (77.4%) followed by Midwakh (15.0%), shisha (6.8%), Cigar (0.66%) and 6.0% smoked more than one type of tobacco product. Out of all smokers in the cohort, 96% were men and 4% were women. Midwakh was smoked more commonly by men and rarely by women.

**Table 2:** Characteristics of tobacco smokers by type of tobacco smoked.

Variable	Cigarettes	Midwakh	Shisha	Cigar	overall
Prevalence	8.55%	1.66%	0.76%	0.07%	11.04%
Prevalence in men	18.77%	3.64%	1.67%	0.16%	24.25%
Proportion of smokers	77.4%	15.0%	6.88%	0.66%	100.0%
Mean age of onset (SD)	22.4 (8.2)	20.9 (6.7)	23.9 (8)	23.5 (8.4)	22.2 (7.8)
Mean Age of Smokers (95% CI)	33.19 (32.9–33.4)	30.05 (29.6–30.4)	31.4 (30.8–32)	34.71 (33.1–36.2)	32.8 (32.6–32.9)
Mean Duration of smoking Years (SD)	11.39 (8.68)	9.34 (7.48)	7.58 (7.15)	11.0 (8.8)	10.84 (8.66)

Table 2 presents the mean duration of smoking for cigarettes, Midwakh, shisha and cigars which were 11.4, 9.3, 7.6 and 11.0 years, respectively. The mean age (SD) of the study participants was 35.2(13.8) years with 74,421 (44%) men and 95,678 (56%) women, whereas the mean age (SD) of smokers was 32.8 years (11.1), 32.7 years (11.1) in men and 35.7 years (12.1) in women. The mean age (SD) of Midwakh smokers was 30.1 (9.4) years, and was lower than that of smokers of all other types of tobacco (mean age 33.2 years, SD 11.2), p<0.001.

**Figure 2:**
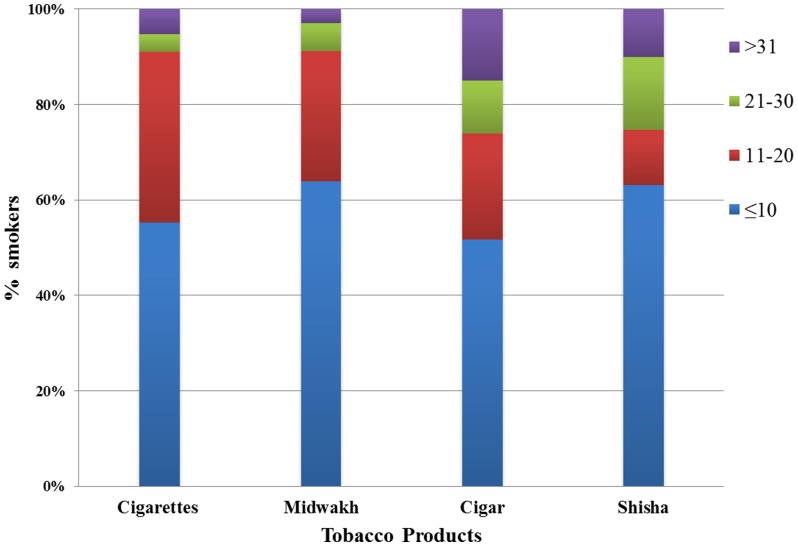
The quantity of different types of tobacco smoked. The quantity described for cigarettes and midwakh is number per day, for cigar smoking is number per week and for shisha smoking is number per month.

Figure 2 shows the quantity of different types of tobacco smoked. Most of the smokers smoked less than 20 per day. On average (SD) people who smoked cigarettes smoked 13.4 (11.5) cigarettes a day and people who smoked Midwakh smoked 12.1 (10.9) a day. On average (SD) people who smoked cigar smoked 18 (19.3) cigars smoked a week and people who smoked shisha smoked 15.6 (21.8) shisha a month.

## Discussion

This is the largest comprehensive survey of tobacco use amongst adults in the UAE and also the largest survey of its kind in the Arab world. It shows that whilst cigarette smoking amongst adult Emiratis is less prevalent than in most other Arab countries [1], the use of Midwakh is now the second most common type of tobacco smoking in the UAE.

The UAE is a relatively young country that was established in 1971 [17] and cigarette smoking was not common amongst UAE nationals 40 years ago. This explains the current low prevalence of smoking, lung cancer and COPD amongst older Emiratis [18], [19]. However after the discovery of oil, the UAE experienced very rapid growth that resulted in massive migration of an expatriate workforce. [20]. As a result of this and the open economy, the consumption of cigarettes and other forms of tobacco smoking has increased.

This study has shown that the average use of Midwakh is 12 times per day which is the equivalent of smoking 6 grams of Dokha (dry tobacco) a day. There is very little in the published literature about the prevalence of Midwakh use in any population with the only previous report being one small cross-sectional survey of university students in Ajman in the UAE that showed 11.5% of the students had smoked Midwakh during their lifetime [6]. Our study shows Midwakh smokers are relatively younger and started smoking at an earlier age than other types of tobacco smokers. Many young smokers prefer Midwakh as it can be used with flavored tobacco and can be used with different pipe styles. Furthermore, smoking Midwakh is cheaper than cigarettes as a week's supply of Dokha for an average smoker only costs $3USD compared to $21 USD for the average cigarette smoker. Although shops are required to check the buyer's age before selling cigarettes, this is not usually practiced for the sale of dry tobacco (Dokha) [7]. This study has the significant strength of being highly representative due to it being population-based with a very large sample size and high response rate (94% of adult Emiratis), so reducing the risk of selection bias. However, it is limited by the fact that it employed self–reported information only with no validation using bio-chemical testing such as cotinine assays. This has been shown to underestimate the true prevalence of smoking [21]–[23]. This may be a particular issue with women as they may feel that it is socially unacceptable for them to admit to smoking [24]. Nevertheless, self-reporting is the commonest method used to report smoking prevalence rates globally [8]–[10].

This study has identified that Midwakh is the second commonest form of tobacco use in the UAE and so questions about its use should be included in all major tobacco surveys for the region. This is particularly important as there is no data on the prevalence of Midwakh smoking in other countries in the region. Although its use has historically been limited to the UAE and Qatar there is anecdotal evidence that it is spreading. The very large expatriate populations in the Gulf may also encourage the spread of the habit to other countries in the region and beyond, as was seen with the rapid spread of shisha smoking over the last twenty years [5], [25].

The UAE federal law on tobacco control which was issued in 2009 forbids smoking (inclusive of Midwakh and shisha) in public places. However, this law has not yet been implemented [26], although municipal authorities are encouraged to enforce the tobacco control law in order to reduce the uptake of tobacco smoking. The law also stipulates health warnings including text and pictorial messaging on tobacco products. Given our findings on the use of Midwakh, it is essential that these are added to Dokha packages to alert users to its harmful effects. The law also needs to make clear that shops will face the same penalties for selling Dokha to minors as they would for cigarettes. Screening for cardiovascular risk in the Weqaya Program is planned at 3-yearly intervals, the data from which will enable us to monitor whether the prevalence of Midwakh use will be reduced after the implementation of the law.

Although the prevalence of tobacco smoking in the UAE is lower than many other countries in the Middle East [27], it is a common habit amongst younger Emiratis and will inevitably lead to an increase in smoking-related comorbidities in the coming decades. The use of Midwakh is of particular concern as is the possibility of the habit spreading to other countries. Local people need to be educated about the harmful effects of smoking Midwakh and it should be included in all tobacco control policies in the region. Further studies on the prevalence of Midwakh use and its health effects are also needed.
